# Self-reported influence of monetary grants in the choice of a medical residency in remote or under-served areas

**DOI:** 10.1186/s13584-018-0272-6

**Published:** 2019-02-15

**Authors:** Yishay Wasserstrum, Racheli Magnezi, Ofer Tamir, Stav Koren, Dor Lotan, Arnon Afek

**Affiliations:** 1 Internal Medicine Department “T”, Chaim Sheba Medical Center in Tel-Ha’Shomer, Ramat-Gan, Israel; 2 Department of Management, Bar Ilan University, Ramat Gan, Israel; 3 Baruch Padeh Medical Center in Poria, Tiberias, Israel; 4 Goldman School of Medicine, Ben Gurion University, Be’er Sheva, Israel; 5 Sackler School of Medicine, Tel Aviv University, Tel Aviv, Israel

## Abstract

**Objectives:**

To evaluate the effect of monetary grants on young physicians’ choice of remote or rural hospital-based practice.

**Background:**

In late 2011, The Israeli Ministry of Health attempted to address a severe physician maldistribution, which involved severe shortages in remotely-located institutions (RLI). The policy intervention included offering monetary grants to residents who chose a residency program in a RLI.

**Methods:**

A total of 222 residents from various disciplines were recruited; 114 residents from RLI and 108 residents from central-located institutions (CLI), who began their residency during 2012–2014. Participants were surveyed on demographic, academic and professional data, and on considerations in the choice of residency location.

**Results:**

Residents in RLI attributed significantly more importance to the grant in their decision-making process than did residents from CLI. This effect remained significant in a multivariate model (OR 1.65, 95% CI 1.20–2.27, *p* = 0.002). The only parameter significantly associated with attributing importance to the grant was older age (OR 1.09, 95% CI 1.00–1.19, *p* = 0.049).

**Conclusion:**

The choice of a RLI for residency may be influenced by monetary grants. This is consistent with real-life data showing an increase in medical staffing in these areas during the program’s duration. Further studies are needed to determine causality and physical practicality of such programs.

**Electronic supplementary material:**

The online version of this article (10.1186/s13584-018-0272-6) contains supplementary material, which is available to authorized users.

## Background

Reducing gaps in the quality and availability of medical services between urban and rural areas, as well as central and remote communities, remains a prime challenge for public health system leaders worldwide. This is an issue of concern to healthcare administrators and regulators, and it affects human rights and social equality. One key component to solving these problems is recognizing the places within the system that suffer from manpower shortages, and require interventions that can alter the balance of supply and demand in their favor. For the purpose of this study, we defined the participating medical centers using a dichotomous definition of central- and remotely-located institutions (CLI and RLI, respectively).

### Existing models to steer physicians to remote or rural regions

It has been shown that deficits in medical manpower affect remote regions first and more strongly than central areas [1]. Nevertheless, simply adding more physicians to the system is a costly intervention that requires a robust infrastructure of academic clinical resources and training facilities, takes many years to begin influencing actual medical staffing and may not be effective in steering physicians to remote regions [2].

Several previous papers have shown that steering physicians towards remotely-located practices through regulatory constraints may have short-term benefits, but a voluntary move would result in better long-term retention rates [3, 4]. In some situations, lifting regulations might harm the effort to staff remotely located practices and RLI, as seen when residents in Japan were given the opportunity to choose their location of residency rather continue mandatorily in the medical centers affiliated to their medical schools [5, 6].

Loan repayment programs that offered retrospective coverage of student loans may play a role in influencing practitioners leaning towards the choice of rural practice [7]. Financial obligations or commitment to a loan forgiveness program may influence recruitment to rural practice [8]. It has been suggested that multi-faceted strategies that address vocational needs alongside financial incentives, have better long term impact on manpower retention [9, 10].

### Predisposing factors for choosing a remotely-located practice

Factors associated with the choice of RLI included personal or spouse’s origin from a remote community, having undergone positive experiences in such a setting during medical school and choice of a medical specialty more compatible with a remote or rural practice, such as primary care [3, 8, 11]. Surprisingly, the role of educational experiences may even surpass that of origin or a desire to live among family and friends [12]. There is a growing global trend toward rural medical schools or programs, which has shown some benefit in physician recruitment and retention, although these reports focus mainly on community based primary care, and not hospital-based practice [13–20]. It has also been suggested that ethnicity, particularly belonging to a visible minority, may be a factor in the choice of practice location [12].

There is evidence that financial considerations play a role in the choice of residency [21]. Nevertheless, in the context of a systemic financial crisis, considerations regarding short-term financial rewards may fall back behind those regarding job security and occupational opportunities [21]. It should also be considered that the choice of moving out of an urban metropolitan area may be, in part at least, from an ideological motive. This may be a problem, as an external reward may impede actions resulting from internal motivation [22, 23].

### The direct monetary grant offered to residents in Israel

In an attempt to address the issue of intranational gaps in medical manpower deployment in Israel, the Israeli Ministry of Health has been running a long-term multi-faceted intervention program. It began with opening a new school of medicine in a northern remote region, and with increasing class sizes in existing classes and opening new programs within existing schools. In late 2011, a new collective agreement for physicians in the Israeli public health system was signed. This agreement included several elements that targeted gaps in periphery-based practices. This included specific budgets that were allocated for increasing physician salaries and adding medical manpower throughout the public hospital system, and specifically residents. These budgets were distributed in a manner that intentionally favored RLI over CLI, thus aiming to close gaps in the numbers and quality of residents in these institutions. Among these means, a specific program providing a one-time monetary grant to residents choosing a residency program in a RLI or in specific disciplines that were defined as having a dire manpower shortage, stood out.

Residents in RLI were given one-time grants as high as 300,000 or 500,000 NIS (at the time, 81,508 or 135,847 USD, respectively), depending on the field of residency. This was equivalent to 115–193% of the estimated minimum annual salary for residents [24, 25]. The main fields included in this program were internal medicine, anesthesiology, neonatology, geriatrics, general and neonatal intensive care, pathology, emergency medicine and physical medicine and rehabilitation. Residents were given the lower sum grant for either choice of RLI residency or a specific field targeted by the program, and the full-sum grant for choice of a RLI-based residency in one of those fields. The grants were given to RLI residents from all disciplines during the years 2012–2014, and concurrently, a drastic rise in physician numbers was observed throughout the entire public hospital system, and more so in remotely-located practices and RLI [26, 27]. The 2011 agreement also had differential salary components. While all residents were given a 32% salary raise spread over 8 years, RLI residents were given an additional 25% raise [24].

## Objectives & assumptions

We sought to evaluate the role of direct monetary grants offered to residents from all medical fields, who chose a residency program in remotely located medical centers, and to understand how financial considerations perform when compared to factors known to traditionally effect the choice of remote of rural practice, such as having a rural community of origin or having undergone clinical rotations in such settings during medical school or an internship program.

## Methods

### Study population and definitions

We performed a retrospective survey of residents from a variety of specialties, recruited from 2 CLI and 6 RLI across the country. The CLI chosen are 2 general hospitals located in the central district. The chosen RLI are 6 of the 7 general hospitals where the grants were given to residents. The last institution is a very small local facility, in which most young physicians are employed on a temporary basis before transferring to a bigger central facility, and thus was not included in the study. Two centers, 1 RLI and 1 CLI were facilities defined as national centers, and the rest were either secondary or local facilities. All participating medical centers were academically affiliated to one of the national faculties of health sciences and their school of medicine at the time of recruitment.

Participants were recruited voluntarily, during a period of several months from September 2014 to September 2015. Where available, forms were disseminated via the local internal mailing systems and participants were asked to fill out the forms and return them to a local administrator. When the internal mailing systems were unavailable to the researchers, the forms were distributed locally in departments by the chief resident, and then returned as a bulk to the authors.

The inclusion criteria for participants in the study were: (1) Residents of all ages, genders and ethnicities; and (2) Current residents in any program in a public medical center. The exclusion criteria were: (1) Residents who began their current residency before January 1st, 2012 or after December 31, 2014; (2) Residents who participated in the pilot phase of this study; (3) Foreign residents during clinical rotations; and (4) Forms missing large amounts of information.

### Study tool

All participants completed a form surveying demographic, academic and professional data, as well as a 5-point Likert scale questionnaire regarding specific considerations in the choice of residency location. The questionnaire was based on a scale from 1 (“Do not agree at all”), through 5 (“Strongly agree”). Regarding the significance of the grant in the choice, the question was phrased as following: “The grant given to physicians who move to peripheral locations was taken into account in the choice of location for my residency”. Prior to building the form, we formally interviewed 10 residents from RLI and 10 residents from CLI, in order to try to make sure that our study tool covers the mainly noticeably significant issues of residency choice.

Prior to distribution, the form was given to 8 independent reviewers, including native Arabic and Russian speakers, for feedback on the clarity of the language used. All forms were distributed to residents via locally available means, mainly internal mailing systems.

### Sample size

Sample size was determined using the Epi Info program, version 6 (CDC, 2011), based on a power of 1-β  =  80% and a *p*-value of α  =  0.05. Based on previously available data [8], we estimated that comparing the resident groups would require a cohort of 212 participants divided equally between both groups to detect a difference of 18% in grant receptivity.

### Statistical analysis

Variables were described according to their properties. Categorical variables are reported in frequencies and percentages, and significance was assessed using the chi-square test or Fischer’s exact test. Continuous variables with a normal distribution were reported as mean and standard deviation values, and significance was assessed using the t-test and ANOVA methods. Continuous variables that did not have a normal distribution were reported as median and interquartile range (IQR, 25th–75th percentiles) values, and significance was assessed using non-parametric Mann-Whitney U and Kruskal-Wallis tests. Likert scale scores are quasi-numeric variables, and as such we treated them according to more strict definitions as a nonparametric variable [28].

Potential covariates were analyzed in univariate logistic regression models, using either a dichotomous evaluation of the grants’ importance in choice of residency location, and of the actual residency location as the dependent variables. Variables found to be significant were further analyzed in a multivariate model with the use of best-subset regression modeling.

All statistical tests were 2-sided, and a *p* value of less than 0.05 was considered significant. The *p* values for interaction are reported. Analyses were carried out with the use of SPSS software, version 23 (IBM Inc.).

## Results

A total of 222 residents from a variety of fields answered the questionnaire at an estimated participation rate of 30%. Participants were classified according to their current location of practice, with a total of 114 residents from RLI and 108 residents from CLI. Personal, academic and professional information of these 2 groups are described in Table 1.

**Table 1:** Study population demographics and academic background

		CLI (*n* = 108)	RLI (*n* = 114)	
		Mean (SD)		*p*
Age (years)		32.3 (±2.8)	32.4 (±4.5)	0.798
		n (%)		
Male gender		59 (55)	83 (74)	0.005
Personal status	Single	32 (30)	30 (27)	0.638
	Married/long term commitment	75 (70)	81 (73)	
Financial background ^A^	Below average	5 (5)	14 (12)	< 0.001
	Average	37 (36)	64 (56)	
	Above average	48 (47)	30 (26)	
	Significantly above average	12 (12)	6 (5)	
Community of origin	Central	77 (71)	18 (16)	< 0.001
	Remote	14 (13)	67 (59)	
	Immigrant	9 (8)	12 (11)	
Spouse’s community of origin	Central	55 (74)	16 (23)	< 0.001
	Remote	10 (14)	44 (62)	
	Immigrant	9 (12)	11 (15)	
Medical school in Israel		58 (56)	29 (26)	< 0.001
Remotely-based clinical rotations during training		57 (53)	89 (78)	< 0.001
Internship in a RLI		10 (10)	68 (65)	< 0.001
Internship and residency in the same institution		38 (35)	47 (42)	0.355
Residency in a field with dire manpower shortage		54 (50)	60 (54)	0.596
Stage of decision on current residency	Before clinical studies	14 (14)	11 (10)	0.406
	Clinical studies	29 (30)	24 (23)	
	Internship	30 (31)	42 (40)	
	Post-internship	24 (25)	29 (27)	

(A) The single participant who responded “significantly below average” was counted as “below average”

RLI residents were more of male gender, had a lower self-reported financial status prior to medical school, had more either self or spousal remote community of origin, more studied in foreign-based medical schools and had more exposure to practice in remote settings during clinical rotations or the mandatory year of internship.

### Attitudes toward the grant depending on resident location

**Fig. 1:**
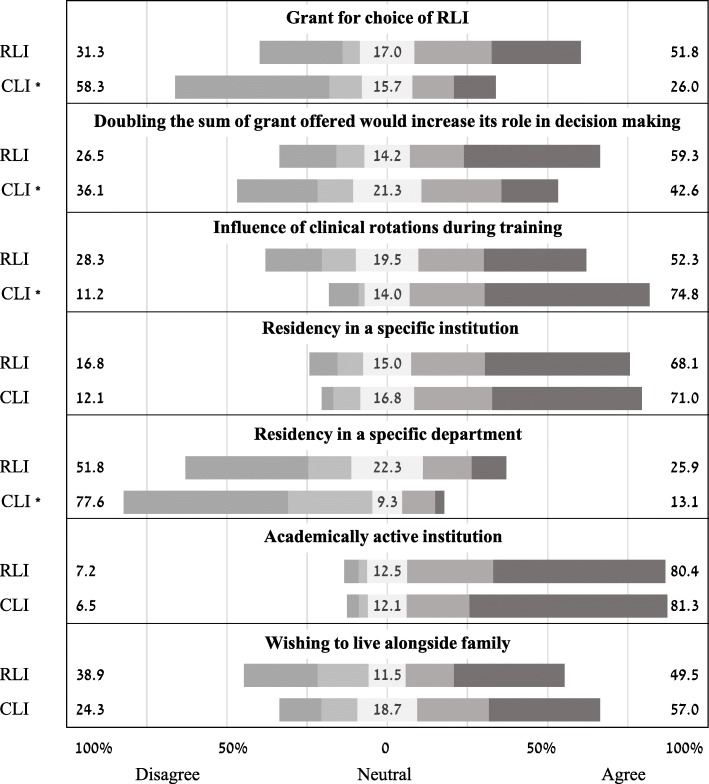
Likert scale ranking of significant factors in choice of residency location

Results of a 5-stage Likert scale of factors in the choice of residency location are shown in Fig. 1. RLI residents attributed higher importance to the grant compared to CLI residents, with 51.8% either agreeing or strongly agreeing that the grant had a role in their decision making, compared to 26.0% in CLI residents. In contrast, 48.1% of the CLI group reported that they strongly disagreed that the grants had a role in their decision-making process, and along with participants that answered they disagree, the total disagreement rate was 58.3%. In RLI residents, these figures were 25.9 and 31.3%, respectively (U = 4068.5, *p* < 0.001). When asked whether a grant of twice amount offered would be a more significant factor in their decision-making process, the RLI resident group remained dominant in its significantly higher agreement, 59.3% Vs. 42.6% for overall agreement, and 42.5% vs. 17.0% for strongly agreeing, respectively (U = 4553.5, *p* = 0.001).

Other considerations presented in the questionnaire that differed significantly were a higher importance attributed to the influence of clinical rotations during training (medical school and internship) in the CLI group (U = 4492.5, *p* = 0.001) and a higher reported desire for a residency in a specific department among the RLI resident group (U = 4683.5, *p* = 0.003).

**Fig. 2:**
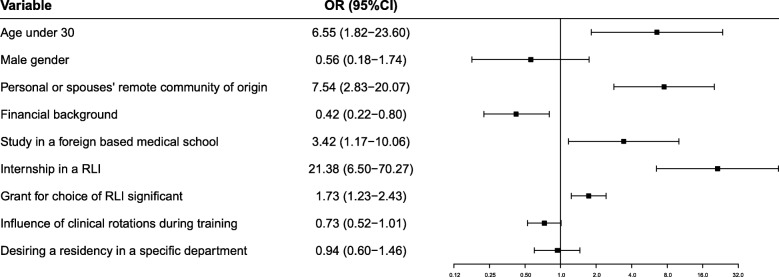
Multivariate analysis for factors associated with the choice of a remote residency location

Figure 2 describes the adjusted model for predictors for the choice of RLI residency. The model was adjusted for covariates that had a *p* value < 0.1 in a univariate model, as well as age and gender. In this model, attribution of importance to the grant given for the choice of a RLI for residency was statistically significant among RLI residents (OR 1.73, 95% CI: 1.23–2.43, *p* = 0.002). Other covariates that remained significantly associated with the RLI group included age under 30 years, a personal or spouse’s origin from a remote community, a weaker financial background, having studied in a foreign-based medical school and having undergone an internship program in a RLI. The full uni- and multivariate models are shown in Additional file 1: Table S1.

### Factors associated with receptivity towards the grant

When examining correlations between different considerations in the choice of residency location within the entire resident population, as calculated using Spearman’s rank correlation coefficient and stratified according to residency location, there was a strong correlation between agreeing that the grant was an important factor and that doubling it would increase its importance (*r* = 0.711, *p* < 0.001), and this effect persisted in both sub-groups with a stronger correlation among RLI residents (*r* = 0.744 and *r* = 0.595, *p* < 0.001 in both cases). Among RLI residents, no other consideration was significantly associated with attribution of importance to the grant in the decision-making process, or to the possibility that it would be more important if doubled among the RLI residents. Among CLI residents, a desire to undergo the residency in a specific department was negatively associated with attribution of importance to the grant (*r* = − 0.211, *p* = 0.03). Also among CLI residents, the possibility that doubling the grant amount would increase its importance was associated with attribution of importance for living in proximity to family (*r* = 0.213, *p* = 0.029).

**Fig. 3:**
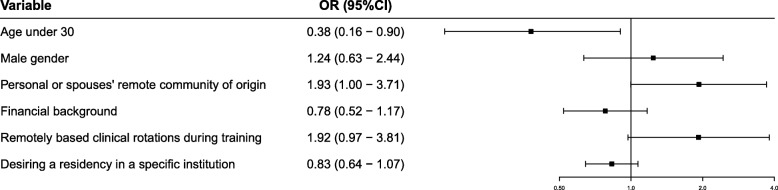
Multivariate analysis for factors associated with the choice of a remote residency location

In order to perform a logistic regression model assessing the receptivity towards the offered grant, we created a dichotomous classification of attitudes towards the grant. Receptive participants were defined as those who responded either “5-Strongly agree” or “4-Agree” to the question regarding the grant, and non-receptive were the remaining participants who responded either neutrally or negatively. The multivariate logistic regression model assessing independent predictors of receptivity towards the grant is described in Fig. 3. The model was adjusted for covariates that had a *p* value under 0.1 in a univariate model, as well as age and gender. In this multivariate model, age under 30 years was significantly predictive of a lower receptivity towards the grant (OR 0.38, 95% CI: 0.16–0.90, *p* = 0.02), and personal or spouse’s origin from a remote community and having undergone clinical rotations during training had borderline significance, as described. None of the other covariates remained significant in the multivariate analysis. The full uni- and multivariate models are shown in Additional file 2: Table S2.

## Discussion

Remote areas may have difficulties attracting medical personnel. This may be connected to professional and academic considerations, as well as to personal considerations such as spousal employment opportunities, weaker social infrastructure (such as education systems) and moving away from familial support system. Although definitions such as *rural, remote* or *periphery* have vastly different meanings in different countries, we believe that there is also a significant component of the public perception and attitudes, that gives a common practical definition to these terms. This may, to some extent, be applicable both in a large country such as Canada or Australia, as well as in a smaller place such as Israel. Nevertheless, this of course is still far from rendering Kenora, Ontario and Tiberias, Israel as more of the same. Another issue is institution size - not all RLI are small, some may be even a tertiary center which is academically affiliated. This means that these institutions may face vastly different challenges in physician recruitment, a disparity that was not addressed in the manner that the grants studied in this paper were offered.

Direct monetary grants are a simple, structured form of incentive. For young physicians, it has the potential to influence key choices early in their professional career and thus enable regulators to steer medical personnel toward regions that otherwise have difficulties attracting medical personnel. As such, its main pitfall is that grant eligibility was based solely on the choice of residency location, thus potentially allocating resources towards subjects who would not otherwise require an extra incentive in order to make the desired decision.

This study investigated a single component of a complex, multi-staged intervention, and therefore it may be argued that some of our findings may be better explained by factors other than the grants, including a general increase in the number of new residents during this period. Published data regarding the gross numbers and rates of residency program enrollment from the years prior to and early years after this program was implemented shows a general increase in the number of new residents nationwide, with a higher increase in RLI. When comparing data from 2008 to 2010 to data from 2012 to 2014 (our study population), the annual rate or new residents in RLI rose from an 17 to 22% [29]. Therefore, it could be that one or more of these other factors, especially the increase in the number of new medical licenses issued annually, may serve as alternative explanations to the observed reality of a general increase in RLI residents.

Nevertheless, the fact that attribution of importance to the grant was independently associated with the choice of a RLI for residency may at least partially answer these concerns. Our model is also compatible with the major factors associated with choice of a remote practice described previously [3, 8, 11]. Furthermore, these factors were not independently associated with a positive attitude towards the grant. We suggest that these strengthen our findings and at least partially overcome the limitations of a model based on retrospective, self-reported data.

Interestingly, 26% of CLI residents either agreed or strongly agreed that the grants for work in the periphery were a significant in their decision-making process. One interpretation to this finding is that these respondents considered moving to the periphery because of the incentive created by the grants, but there were other considerations or personal limitations that overpowered the influence of the grant. Another possibility is that this reflects a certain bias in the self-reporting of personal considerations, as many participants were reluctant to admit to dismissing the opportunity to receive a large financial incentive. Due to the other socio-economic factors that are not solved by grants or other elements of intervention implemented during the study period as mentioned above. we believe that the prior is a more predominant explanation for this finding than the latter. Also worth noting, is that although factors shown to be associated with the choice of RLI in previous studies were also significantly associated with choice or RLI in this study, without being significantly associated to receptivity towards the grant offered. This may serve as evidence of a diminished role for confirmation bias among respondents.

The relationship between the personal factors we surveyed suggests that financial considerations may have a dose-dependent effect in regard to the offered sum, which is not at all intuitive when discussing grants that are well above the regular annual salary of these residents. The relative lack of significant correlations between the various factors suggests that for an individual, the composition of factors and the level of their importance vary and may be more reflective of personal needs and points of view.

The main concern regarding financial incentives is that of cost-efficacy. Incentivizing all physicians who choose to practice in remote regions means that some of the funds allocated will go to those who would have made that choice anyway. Incentives given according to a differential may be more cost-effective, yet this poses a different challenge, as these might cause negative emotions in non-eligible workers and raise workplace tensions.

Studies examining financial incentives outside the medical community show that these incentives may not be the optimal solution for attracting manpower towards career choices perceived as less attractive [22, 30, 31]. Furthermore, it has been suggested that in certain cases, such incentives may even have paradoxical effects in which extrinsic rewards weaken motivation generated by intrinsic motives. This may lead to decreased productivity, especially if the incentive is small in a way that may reflect a lesser perception of the targeted behavior [30]. It was also suggested that external incentives may decrease willingness to perform tasks that are perceived as having a higher social value [22, 23]. These effects have been associated with female gender, which may become more significant as the prevalence of female physicians continues to increase [31, 32].

## Limitations

Our study has several inherent limitations. First, since the grants were offered indiscriminately, there was no way for us to create external control groups. In addition, the use of retrospective self-reported importance left us vulnerable to attribution and recall bias, and our findings should be considered accordingly. This of course limits the applicability of our findings in terms of policy implication, and although this is a positive signal, more prospective and robustly designed studies in this field are warranted in order to better solidify our findings. Also, in the absence of central or local databases regarding application for residency programs and acceptance rates, we could not obtain data confirming that the current residency program for our participants was their original preference, and not a reluctant choice. There is also concern that the 2 medical centers chosen for the CLI cohort may not be representative of the CLI population in its’ entirety.

As shown in Table 1, the study groups vary significantly in key traits. We have attempted to attenuate these differences by using a multivariate regression model and propensity score adjustment, although from a statistical view, our model is weakened by the lack of ability to perform matching (i.e by propensity score matching etc.).

We were also unable to acquire data regarding non-respondents, and thus are unable to rule out any selection bias. Thus, the study sample may not be representative of the population we set out to study. The low response rate of ~ 30% raises concerns regarding the representativeness of our cohort.

## Conclusion

Direct monetary grants may be a simple and useful tool in rapid intervention programs attempting to direct medical personnel towards otherwise less attractive parts of a healthcare system. Further studies are needed to assess the cost effectiveness of such programs, as well as the relationship between grants and other possible intervention measures that may be taken concomitantly. Further study is also needed to better define the optimal size of grants offered.

## Additional files

Additional file 1: **Table S1.** Multivariate analysis for the choice of a remote residency location. (DOCX 17 kb)

Additional file 2: **Table S2.** Multivariate analysis for self-reported receptivity to a grant for choosing a remote location for residency. (DOCX 16 kb)
