# Supplementary material for: Self-reported influence of monetary grants in the choice of a medical residency in remote or under-served areas
Source: Isr J Health Policy Res. 2019 Feb 15;8:1. doi: 10.1186/s13584-018-0272-6 (PMC6376660; doi:10.1186/s13584-018-0272-6)
Supplement: Supplementary file 1 — Table S1. Multivariate analysis for the choice of a remote residency location. (DOCX 17 kb) [file 13584_2018_272_MOESM1_ESM.docx]

| **Table S1. Multivariate analysis for the choice of a remote residency location** | | | | | | | |
| --- | --- | --- | --- | --- | --- | --- | --- |
|  | **Univariate model** | | | | **Multivariate model** *^B^* | | |
|  | **n** | **Odds ratio** | **95% CI** | **p** | **Odds ratio** | **95% CI** | **p** |
| **Age over 30** | 217 | 0.60 | 0.33 - 1.09 | 0.092 | 6.55 | 1.82 - 23.60 | 0.004 |
| **Male gender** | 219 | 2.33 | 1.32 - 4.11 | 0.004 | 0.56 | 0.18 - 1.74 | 0.313 |
| **Personal status, married** *^A^* | 218 | 1.12 | 0.64 - 2.08 | 0.638 |  |  |  |
| **Personal or spouses' remote community of origin** | 203 | 10.07 | 5.27 - 19.25 | <0.001 | 7.54 | 2.83 - 20.07 | <0.001 |
| **Immigrant** | 220 | 1.67 | 0.70 - 3.99 | 0.251 |  |  |  |
| **Financial background** | 216 | 0.48 | 0.32 - 0.70 | <0.001 | 0.42 | 0.22 - 0.80 | 0.008 |
| **Study in a foreign-based medical school** | 216 | 3.61 | 2.03 - 6.40 | <0.001 | 3.42 | 1.17 - 10.06 | 0.025 |
| **Remotely-based clinical rotations during training** *^C^* | 222 | 3.19 | 1.78 - 5.70 | <0.001 |  |  |  |
| **Internship in a RLI** | 205 | 16.54 | 7.69 - 35.59 | <0.001 | 21.38 | 6.50 - 70.27 | <0.001 |
| **Residency in a field with dire manpower shortage** | 220 | 0.87 | 0.51 - 1.47 | 0.867 |  |  |  |
| **Stage of decision on current residency** | 203 | 1.21 | 0.91 - 1.60 | 0.191 |  |  |  |
| **Grant for choice of RLI significant** | 220 | 1.48 | 1.24 - 1.77 | <0.001 | 1.73 | 1.23 - 2.43 | 0.002 |
| **Influence of clinical rotations during training** | 220 | 0.71 | 0.58 - 0.86 | 0.001 | 0.73 | 0.52 - 1.01 | 0.057 |
| **Desiring a residency at a specific institution** | 220 | 0.92 | 0.74 - 1.34 | 0.458 |  |  |  |
| **Desiring a residency in a specific department** | 219 | 1.42 | 1.15 - 1.77 | 0.001 | 0.94 | 0.60 - 1.46 | 0.770 |
| **Abundant academic activity available** | 219 | 0.92 | 0.71 - 1.19 | 0.524 |  |  |  |
| **Wishing to live near family** | 220 | 0.87 | 0.73 - 1.04 | 0.130 |  |  |  |
| (A) Married or in any form of long-term partnership. (B) Model includes age, gender and any covariate with a p-value under 0.1 in the univariate model. (C) Was not included in the multivariate analysis because of overlap with the stronger covariate of internship in a RLI. | | | | | | | |
