# Supplementary material for: Self-reported influence of monetary grants in the choice of a medical residency in remote or under-served areas
Source: Isr J Health Policy Res. 2019 Feb 15;8:1. doi: 10.1186/s13584-018-0272-6 (PMC6376660; doi:10.1186/s13584-018-0272-6)
Supplement: Supplementary file 2 — Table S2. Multivariate analysis for self-reported receptivity to a grant for choosing a remote location for residency. (DOCX 16 kb) [file 13584_2018_272_MOESM2_ESM.docx]

| **Table S2. Multivariate analysis for self-reported receptivity to a grant for choosing a remote location for residency** | | | | | | | |
| --- | --- | --- | --- | --- | --- | --- | --- |
|  | **Univariate model** | | | | **Multivariate model** ^B^ | | |
|  | **n** | **Odds ratio** | **95% CI** | **p** | **Odds ratio** | **95% CI** | **p** |
| **Age over 30** | 215 | 1.61 | 0.86 - 2.99 | 0.134 | 0.38 | 0.16 - 0.90 | 0.028 |
| **Male gender** | 218 | 2.02 | 1.11 - 3.66 | 0.021 | 1.24 | 0.63 - 2.44 | 0.527 |
| **Personal status, married** ^A^ | 216 | 1.43 | 0.77 - 2.64 | 0.259 |  |  |  |
| **Personal or spouses' remote community of origin** | 201 | 2.15 | 1.21 - 3.84 | 0.009 | 1.93 | 1.00 - 3.71 | 0.051 |
| **Immigrant** | 218 | 0.99 | 0.41 - 2.39 | 0.974 |  |  |  |
| **Financial background** | 215 | 0.69 | 0.48 - 1.00 | 0.048 | 0.78 | 0.52 - 1.17 | 0.230 |
| **Study in a foreign-based medical school** | 214 | 1.23 | 0.70 - 2.16 | 0.467 |  |  |  |
| **Remotely-based clinical rotations during training** ^C^ | 220 | 1.96 | 1.08 - 3.56 | 0.026 | 1.92 | 0.97 - 3.81 | 0.060 |
| **Internship in a RLI** | 204 | 1.60 | 0.90 - 2.84 | 0.111 |  |  |  |
| **Residency in a field in dire manpower shortage** | 220 | 1.36 | 0.79 - 2.34 | 0.271 |  |  |  |
| **Stage of decision on current residency** | 201 | 1.07 | 0.80 - 1.43 | 0.663 |  |  |  |
| **Influence of clinical rotations during training** | 219 | 0.86 | 0.71 - 1.04 | 0.112 |  |  |  |
| **Desiring a residency in a specific institution** | 219 | 0.78 | 0.62 - 0.97 | 0.025 | 0.83 | 0.64 - 1.07 | 0.150 |
| **Desiring a residency in a specific department** | 218 | 1.07 | 0.87 - 1.32 | 0.508 |  |  |  |
| **Abundant academic activity available at the institution** | 218 | 0.91 | 0.71 - 1.18 | 0.488 |  |  |  |
| **Wishing to live near to family** | 219 | 0.98 | 0.82 - 1.17 | 0.797 |  |  |  |
| (A) Married or in any form of long-term partnership. (B) Model includes age, gender and any covariate with a p-value under 0.1 in a univariate model. (C) Was not included in the multivariate analysis because of overlap with the stronger covariate on any exposure to remote practices during training. | | | | | | | |
